# Identification of factors limiting the efficiency of transplanting extracellular electron transfer chains in *Escherichia coli*

**DOI:** 10.1128/aem.00685-25

**Published:** 2025-05-13

**Authors:** Laura-Alina Philipp, Lukas Kneuer, Carina Mayer-Windhorst, Simon Jautelat, Nhat Quang Le, Johannes Gescher

**Affiliations:** 1 Institute of Technical Microbiology, Hamburg University of Technology 682475, Hamburg, Germany; Michigan State University, East Lansing, Michigan, USA

**Keywords:** synthetic biology, exoelectrogens, *c*-type cytochromes, heterologous gene expression, microbiology

## Abstract

Research in electro-microbiology provides unique opportunities to study and exploit microbial physiology. Several efforts have been made to transplant the extracellular electron transport chain from the native exoelectrogenic model organism *Shewanella oneidensis* into *Escherichia coli*. However, systematic comparisons between donor and recipient strain configurations are largely missing. Hence, the proposed minimal protein set, consisting of the *c*-type cytochromes cytoplasmic membrane protein A (CymA), small tetraheme cytochrome (STC), MtrA, and MtrC, as well as the *β*-barrel protein MtrB, was heterologously expressed in *E. coli* in different expansion stages. These stages were compared to corresponding *S. oneidensis* strains in terms of anthraquinone-2,6-disulfonate (AQDS) and ferric citrate reduction rates. This revealed that transplantation of heterologous extracellular electron transfer (EET) chains is associated with a tremendous decrease in electron transfer rates. As the acquired electron transfer rates were not competitive to *S. oneidensis*, it was hypothesized that protein localization and maturation might be affected by heterologous expression. Hence, the type II secretion system from *S. oneidensis* was also transplanted into an *E. coli* strain. The latter allowed the secretion of the terminal reductase MtrC onto the cell surface of *E. coli* for the first time. This was correlated with significantly increased but still insufficient extracellular electron transfer rates. Further experiments suggest that the correct folding of MtrB might be a further bottleneck.

**IMPORTANCE:**

Research on transplanting extracellular electron transfer (EET) chains into non-native exoelectrogens is vital for advancing bioenergy and bioremediation technologies. Enabling these organisms to transfer electrons to external surfaces like anodes can enhance microbial fuel cell efficiency and electricity generation from organic waste. This approach can broaden the range of substrates and products for biotechnological applications, offering innovative solutions for sustainable production. Our work shows that transplanting the EET chain of *Shewanella oneidensis* into *Escherichia coli* is more complex than previously suggested. The heterologous expression of only *c*-type cytochromes and the β-barrel protein MtrB is insufficient for competitive reduction rates. Predominantly, MtrC and MtrB require specific proteins for transport and folding, necessitating co-expression and maturation. We could identify the type II secretion system of *S. oneidensis* as crucial for MtrC secretion in *E. coli*. Thereby, this work highlights the substrate specificity of bacterial type II secretion systems, suggesting methods to optimize protein production and secretion in bioelectrochemical applications.

## INTRODUCTION

Some microorganisms can transfer respiratory electrons to soluble or insoluble extracellular electron acceptors. Extracellular electron transfer (EET) is an important process. For instance, the electron acceptor iron is the fourth most abundant element in the Earth’s crust and typically occurs as insoluble iron oxides or oxyhydroxides, which can be reduced by the organisms and can then undergo a variety of important abiotic redox reactions. Moreover, EET can be applied in bioelectrochemical systems in which the organisms use a solid-state anode as a surrogate for natural insoluble electron acceptors. Different strategies have evolved in so-called exoelectrogenic organisms to extend the respiratory chain beyond the cytoplasmic membrane for the reduction of extracellular electron acceptors (1–7). Although the ability for EET is not exclusive to Gram-negative organisms, but has also been described for Gram-positive organisms, archaea, microalgae, and fungi, the two proteobacteria *Shewanella oneidensis* and *Geobacter sulfurreducens* are the most widely used and understood model organisms for this process (1, 4, 8–11). From these, the system of *S. oneidensis* is currently understood best.

**Fig 1:**
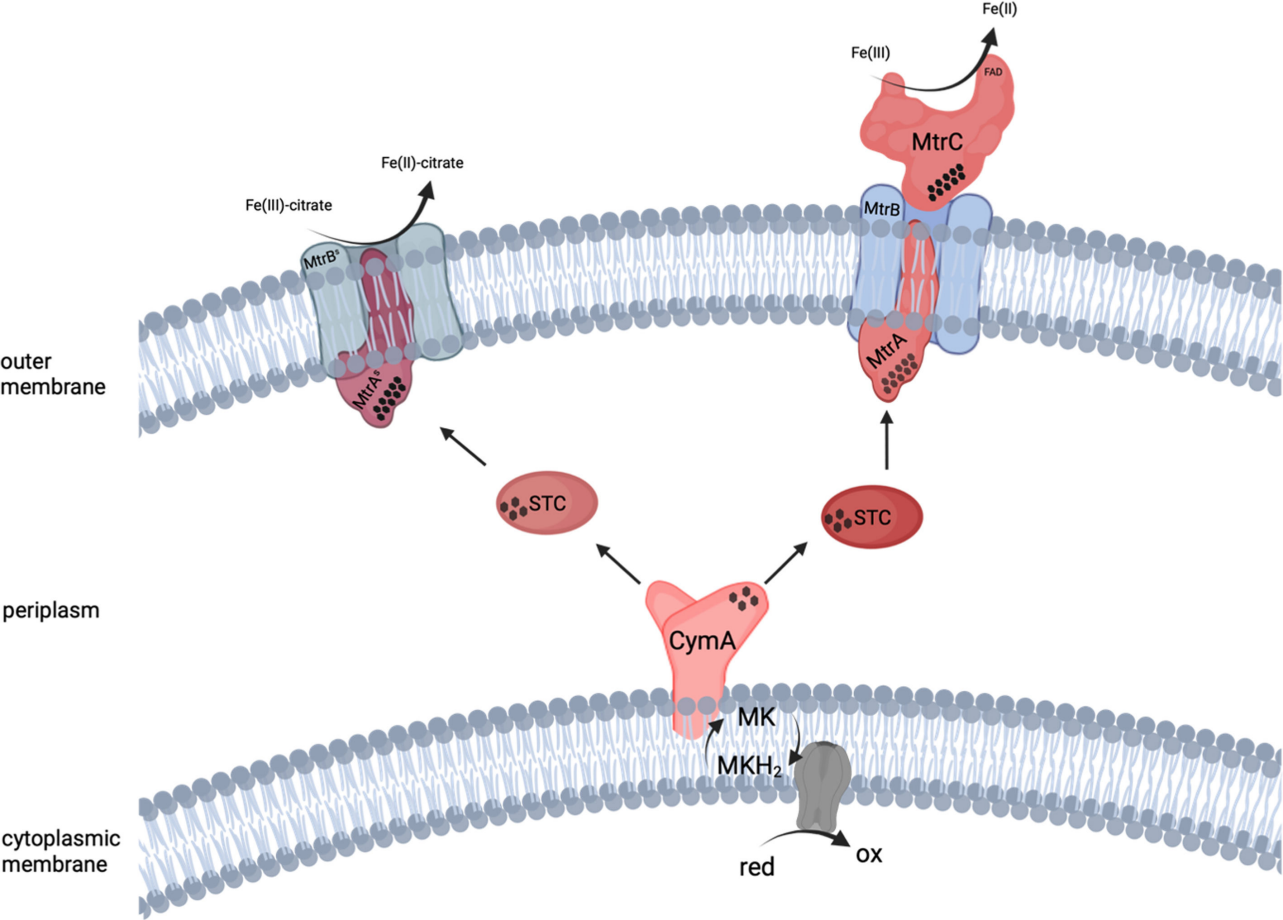
Putative minimal protein set required for EET in *S. oneidensis*. Respiratory electrons are transferred from the menaquinone pool to CymA. Subsequently, electrons are transported through the periplasm via STC and are then transferred to the outer membrane surface via the MtrCAB complex, where (in)soluble electron acceptors are reduced. A suppressor mutant, MtrAB^s^, partially compensates for the loss of MtrC, and soluble extracellular electron acceptors such as Fe(III)-citrate can be reduced in this mutant without the presence of outer membrane cytochromes. Diamonds represent the number but not the arrangement of hemes within each *c*-type cytochrome, and the terms red and ox represent reduced and oxidized intracellular compounds. Figure created using data from references 20, 22.

To reduce extracellular electron acceptors, *S. oneidensis* relies on a network of multiheme *c*-type cytochromes localized in the periplasm and the inner and outer membranes. Several studies identified distinct EET routes to the cell surface and highlighted the functional redundancy of several *c*-type cytochromes. This led to the postulation of a putative minimal set of proteins (Fig. 1) that enables *S. oneidensis* to perform EET (12–21).

According to this putative minimal protein set, electrons from the menaquinone pool are first transferred to the periplasm via the 20.8 kDa CymA, a tetraheme *c*-type cytochrome belonging to the NapC/NirT family (23, 24). CymA is localized in the cytoplasmic membrane via an N-terminal, *α*-helical transmembrane anchor and protrudes into the periplasmic space with a globular tetraheme domain. CymA is not only involved in electron transfer to Fe(III) or an anode, but rather represents the main hub for the distribution of respiratory electrons to diverse electron transport chains. Via these routes, e.g., fumarate, dimethyl sulfoxide (DMSO), nitrate, or nitrite, are subsequently reduced (13, 23, 25, 26). In addition, CymA is necessary for growth with DMSO or nitrite as an electron acceptor (13, 27–32). CymA is not only a quinol dehydrogenase but also seems to use menaquinone-7 as a cofactor (24). Because the 235 Å distance between inner and outer membrane is too wide to allow direct electron transfer, *S. oneidensis* relies on two soluble periplasmic *c*-type cytochromes, small tetraheme cytochrome (STC) (12.9 kDa) and/or fumarate reductase (FccA) (65 kDa), that act as periplasmic electron shuttles (20, 21, 29, 33–35). It was demonstrated that FccA and STC cannot interact with each other due to their negative surface charge, but the two cytochromes may compete for the binding site at MtrA (20). For this study, we chose STC as a periplasmic electron shuttle since in previous studies, *S. oneidensis* Δ*cctA* (the corresponding gene for STC) mutants exhibited slower ferric iron reduction rates, which may be due to slower dissociation of CymA and FccA compared to CymA and STC (20, 29).

Electron transfer across the outer membrane is mediated by a trimeric porin-cytochrome complex that spans the outer membrane and transfers electrons from the periplasmic *c*-type cytochromes STC and FccA to terminal electron acceptors. This so-called MtrCAB complex (mtr: **m**e**t**al **r**educing) consists of the two *c*-type cytochromes MtrC (83 kDa) and MtrA (32 kDa) and the *β*-barrel protein MtrB (76 kDa), which forms a pore in the outer membrane, facilitating interaction between MtrA on the periplasmic side and MtrC on the cell surface (12, 36–40). Since MtrB cannot be detected in *mtrA* deletion mutants, MtrA was suggested to play a chaperone role by assisting in the maturation and integration of MtrB into the outer membrane (38, 41). MtrB is a *β*-barrel protein with 28 transmembrane domains, whereas the crystal structure of MtrB from *Shewanella baltica* suggests that only 26 of the 28 span the outer membrane to form a pore (12, 32, 40). This crystal structure also shows that the pore formed by MtrB tapers toward the cell surface from 30 Å on the periplasmic side to 15 Å on the cell surface, allowing MtrA to enter MtrB but not span into the extracellular space. At the cell surface, MtrA is largely shielded by the extracellular loops of MtrB, leaving only the amino acid residues (AS)284–306 and one of the 10 hemes to interact with MtrC (40). The terminal reductase MtrC is also a decaheme *c*-type cytochrome. MtrC has a lipid anchor through which it is anchored to the outside of the outer membrane after secretion via the type II secretion system (42). MtrC is one of five outer membrane cytochromes (OMCs; MtrC, OmcA, MtrF, SO_1659, and SO_2931) with partially redundant functions (17, 43, 44). In one study, OmcA, another one of these five cytochromes, was found to be loosely attached to MtrC on the cell surface as the second terminal reductase (45). Both *c*-type cytochromes MtrC and OmcA contain flavin cofactors that accelerate the terminal electron transfer step and enable single-electron transport via the formation of semiquinones (46–50). However, OmcA appears to be most important for manganese oxide reduction, as the deletion phenotype for other extracellular electron acceptors is rather negligible (15, 51–53).

In *S. oneidensis* mutants with deletions in genes for all OMCs, a suppressor mutant was also identified that can partially rescue the phenotype of loss of outer membrane cytochromes. In this mutant, amino acid substitutions occurred in both MtrA ((AS)290 Asn→Lys) and MtrB ((AS)219 Asn→Lys). This presumably leads to an altered conformation of both proteins, allowing the suppressor protein MtrA^s^ to project further through the suppressor variation MtrB^s^ into the extracellular space. Reduction assays with the corresponding mutant showed that soluble extracellular electron acceptors can be reduced. In the case of Fe(III)-citrate, the reduction even occurred at near wild-type levels (84%) (22).

Several attempts have already been made to transplant parts or even the whole electron transport chain from *S. oneidensis* into *Escherichia coli* (54–57). This is mostly due to the higher flexibility of *E. coli* regarding usable carbon sources, the wide integration of *E. coli* in biotechnological processes, as well as the fundamental research question of whether we have indeed understood the extracellular electron transport system of *S. oneidensis* well enough to transplant it in another Gram-negative organism. However, so far, none of the approaches has resulted in engineered *E. coli* cells with extracellular electron transfer rates comparable to those of *S. oneidensis*. Although several studies have explored the transplantation of EET chains, directly comparable values are often lacking. Reported current densities for *S. oneidensis* WT vary considerably, ranging, e.g., from 24.0 ± 0.3 µA/cm² (58) to 590 ± 25 µA/cm² (59), depending on factors such as buffer capacity, anode material, anode surface-to-reactor volume ratio, applied potential, and many more. These variations make direct comparisons across studies challenging, further highlighting the necessity of the here-conducted investigation in which direct comparisons between different *S. oneidensis* and *E. coli* strains were conducted to identify potential bottlenecks of the *E. coli* system that had not yet been discovered previously. To this end, we transplanted the complete type II secretion machinery from *S. oneidensis* into *E. coli,* which led to correct MtrC localization. Nevertheless, our study suggests that *E. coli* might still be challenged by its inability to fold MtrB correctly into the outer membrane, a factor that has so far not been identified.

## MATERIALS AND METHODS

### Vector and strain construction

Strains used in this study are listed in Table 1. Relevant genotypes are described for each strain, respectively.

**TABLE 1:** Strains used in this study

Strain	Relevant genotype	Reference
*S. oneidensis* WT	*S. oneidensis* MR-1	(60)
*S. oneidensis* ΔOMCs	*S. oneidensis* MR-1 Δ*OMCs* (all outer membrane cytochromes)	(43)
*S. oneidensis* suppressor	*S. oneidensis* MR-1 Δ*OMCs MtrAB^s^*	(43)
*S. oneidensis* MtrC_snap_	*S. oneidensis* MR-1 pBAD*-mtrC_snap_*	This study
*S. oneidensis* ΔGspD MtrC_snap_	*S. oneidensis* MR-1 Δ*gspD* pBAD*-mtrC_snap_*	This study
*E. coli* WT DH5αZI	*E. coli* DH5αZI *aci q*, PN25‐*tetR*, SpR, *deoR*, *supE44*, Δ(*lacZYAargFV*169), Phi80 *lacZDM15*	(61)
*E. coli* CymA	*E. coli* DH5αZI Δ(*napC-F*) Δ(*frdA-D*)::P_tet__*cymA,* pEC86	(28)
*E. coli* CymA STC	*E. coli* DH5αZI Δ(*napC-F*) Δ(*frdA-D*)::P_tet__*cymA,* pEC86, pMAL-*cctA*	This study
*E. coli* CymA STC MtrAB	*E. coli* DH5αZI Δ(*napC-F*) Δ(*frdA-D*)::P_tet__*cymA* attP21::P_ara_*_mtrAB,* pEC86, pMAL-*cctA*	This study
*E. coli* CymA STC MtrAB^s^	*E. coli* DH5αZI Δ(*napC-F*) Δ(*frdA-D*)::P_tet__*cymA* attP21::P_ara_*_mtrAB^s^*, pEC86, pMAL-*cctA*	This study
*E. coli* CymA STC MtrCAB_1_	*E. coli* DH5αZI Δ(*napC-F*) Δ(*frdA-D*)::P_tet__*cymA* attP21::P_ara_*_mtrAB,* pEC86, pMAL-*cctA-mtrC*	This study
*E. coli* TIISS*_S.oneidensis_*	*E. coli* DH5αZI Δ(*napC-F*) Δ(*frdA-D*)::P_tet__*cymA* KII::P_lac_ _gspHIJK KIII::P_lac_ _*gspGLMN attP21*:: P_lac__*gspDEFC*	This study
*E. coli* TIISS*_S.oneidensis_* MtrC_snap_	*E. coli* DH5αZI Δ(*napC-F*) Δ(*frdA-D*)::P_tet__*cymA* KII:: P_lac__gspHIJK KIII::P_lac__*gspGLMN* attP21::P_lac_*_gspDEFC* pEC86, pBAD-*mtrC_snap__pilD*	This study
*E. coli* DH5αZI MtrC_snap_	*E. coli* DH5αZI pEC86, pBAD-*mtrC_snap_*	This study
*E. coli* BL21 MtrC_snap_	*E. coli* BL21 pEC86, pBAD-*mtrC_snap_*	This study
*E. coli* CymA STC MtrCAB TIISS*_S.oneidensis_*	*E. coli* DH5αZI Δ(*napC-F*) Δ(*frdA-D*)::P_tet__*cymA* KII:: P_lac__gspHIJK KIII::P_lac__*gspGLMN* attP21::P_lac_*_gspDEFC,* pEC86, pMAL-*cctA-mtrC_snap-_pilD,*	This study
*E. coli* DH5αZI MtrAB	*E. coli* DH5αZI pBAD-*mtrAB*	This study
*E. coli* BL21 MtrAB	*E. coli* BL21 pBAD-*mtrAB*	This study
*E. coli* CymA STC MtrCAB_2_	*E. coli* DH5αZI *Δ(napC-F) Δ(frdA-D)::Ptet_cymA* pEC86*_gentR_mtrAB* pMAL*_cctA_mtrCsnap_pilD*	This study

### Construction of *S. oneidensis* and *E. coli* mutants and expression plasmids

Derivatives of pBAD, pMAL-p2E, pAH95, and pEC86 vectors were constructed through isothermal *in vitro* recombination according to reference (62 using primers, listed in Table S1. For heterologous expression of *c*-type cytochromes in *E. coli*, pEC86 was used as described elsewhere (63). All restriction enzymes were obtained from New England Biolabs and used according to the manufacturer’s instructions.

Genomic integration of *gspDEFC* and *cctA,* respectively, was facilitated via CRIM-integration (conditional-replication, integration, and modular plasmid) described by Haldimann and Wanner (64) using the plasmids pAH95 and pAH121. All constructs were verified via Sanger sequencing conducted by Eurofins Genomics and Sequencing. The results were evaluated via the CLC genomics workbench (Qiagen, Venlo).

### Cell cultivation

*E. coli* strains were grown under oxic conditions in lysogeny broth (LB) medium or anoxically in M9 minimal medium (47.8 mM Na_2_HPO_4_, 22 mM KH_2_PO_4_, 9.2 mM NaCl, 18.7 mM NH_4_Cl) supplemented with 0.1 mM CaCl_2_, 1 mM MgSO_4_, trace elements (5 µM CoCl_2_, 0.2 µM CuSO_4_, 57 µM H_3_BO_3_, 5.4 µM FeCl_2_, 1.3 µM MnSO_4_, 67.2 µM Na_2_EDTA, 3.9 µM Na_2_MoO_4_, 1.5 µM Na_2_SeO_4_, 5 µM NiCl_2_, and 1 µM ZnSO_4_), 1 g L^−1^ casein hydrolysate, and 14.8 µM thiamine hydrochloride. The provided carbon source in all *E. coli* minimal medium growth experiments was 0.5% (wt/vol) glycerol. DMSO was used as an electron acceptor at a concentration of 50 mM. Additionally, we added 1 mM Fe-NTA to the medium.

*S. oneidensis* strains were grown in LB medium or anoxically in LB medium with 50 mM HEPES, 50 mM lactate as a carbon source, and 50 mM fumarate. If necessary, ampicillin (100 µg mL^−1^), kanamycin (10 or 50 µg mL^−1^), gentamycin (15 µg mL^−1^), or chloramphenicol (30 µg mL^−1^) was added to the medium.

Heterologous protein expression was induced by adding the appropriate inducers: IPTG 10 µM, AHT 0.43 µM, or arabinose 1 mM. After induction, further incubation was performed at room temperature or at 30°C to ensure optimal protein expression.

### Cell fractionation

For the preparation of membrane fractions, cells were resuspended in buffer (50 mM HEPES, 250 mM NaCl, 0.1 mg mL^−1^ DNase I, pH 7.5) and lysed via French press (1,260 psi). Membrane fractions were obtained via centrifugation at 208,000 × *g* for 1 h and resuspended in the same buffer without DNase I, containing 1%–2% Triton X-100.

### SDS-PAGE and protein visualization

Protein concentrations were determined using Roti-Quant (Roth, Germany) with bovine serum albumin (BSA) as standard. Polyacrylamide gels (12%) were used according to the Laemmli method (65). A total of 100 µg protein per membrane sample and 50 µg protein per periplasmic sample were loaded. Proteins were visualized by Coomassie blue staining (66) using InstantBlue (Expedeon, Germany) according to the manufacturer’s protocol. If necessary, gels were examined for the presence of *c*-type cytochromes prior to Coomassie staining via peroxidase staining as described elsewhere (67).

### Heme quantification

Heme quantification was conducted according to Berry and Trumpower (68). Absorbance was recorded using a Varian Cary 5 spectrometer (Agilent Technologies). The respective sample (250 µL, diluted if necessary) was mixed with 250 µL of a stock solution of 40% (vol/vol) pyridine in 200 mM NaOH and 3 µL of 0.1 M K_3_[Fe(CN)_6_] solution in a 0.5 mL quartz cuvette. The oxidized spectrum (400–600 nm) was recorded. Solid sodium dithionite (approximately 1 mg) was then added stepwise, and the reduced spectrum was recorded after each step until no change in absorbance was obtained by further addition of sodium dithionite. To determine the heme content of the sample, the difference between oxidized and reduced spectra was determined. The calculation was performed according to the formula shown below:


$$A=\left[A_{550}\left(red\right)-A_{550}\left(ox\right)\right]-\left[A_{535}\left(red\right)-A_{535}\left(ox\right)\right].$$

Where *A* = absorbance, red = reduced, and ox = oxidized.


$$c=\frac{A}{\varepsilon \;\cdot \;d}.$$

Where *A* = absorbance, ε = molar extinction coefficient, *c* = concentration, *d* = pathlength absorbance coefficient ε = 24 mM^−1^ cm^−1^ (69).

### Western blot analysis

The samples were heated for 5 min to either 37°C or 95°C with SDS-loading dye (containing 1.9 mM *β*-mercaptoethanol). A 50 µg (*S. oneidensis*) or 100 µg (*E. coli*) protein per sample was loaded on a gel and separated via SDS-PAGE. Proteins were blotted onto nitrocellulose membranes using the TransBlot Turbo blotter with 1.3 A, 25 V for 12 min (Bio-Rad, Germany). Detection was carried out using antibodies binding a specific MtrB epitope (amino acid residues 23–44 [70]) and alkaline phosphatase-coupled anti-rabbit antibodies (AP detection kit; Bio-Rad, Germany).

### AQDS reduction assay

Cells were pre-grown until OD_600_ 0.4–0.6, appropriate inducers were then added, and the culture was incubated at 30°C for an additional 12 h. Cells were harvested and washed twice before the start of the experiments. The assay was performed in an anaerobic chamber (Coy Laboratory Products, MI, USA) by measuring the absorbance of the cultures (OD_600_ = 6) in a volume of 200 µL in triplicate if not stated otherwise at 436 nm over 6 h (1 measurement point/minute) using an infinitePro 200 spectrometer (Tecan). Glycerol in a concentration of 50 mM and 1 mM anthraquinone-2,6-disulfonate (AQDS) served as electron donor and acceptor, respectively.

### Ferric citrate reduction assay

Cells were pre-grown until OD_600_ 0.4–0.6 in M9 minimal medium with 0.5% (wt/vol) glycerol and 50 mM DMSO. Appropriate inducers were then added, and the culture was incubated at 30°C for an additional 12 h. Cells were harvested and washed twice before the start of the experiments. The assay was performed as a cell suspension assay (OD_600_ = 6) in triplicate in an anaerobic chamber (Coy Laboratory Products, MI, USA). A total of 50 mM glycerol and 10 mM Fe(III)-citrate served as electron donor and acceptor, respectively. Re-oxidation of Fe(II) was prevented by adding 100 µL 3 M HCl to 200 µL cell suspension. For quantitative determination of the Fe(II) content, a ferrozine assay according to Stookey was performed (71). Ferrozine (dinatrium-4-[3-pyridin-2-yl-6-(4-sulfonatophenyl)-1,2,4-triazin-5-yl]-benzosulfonate 0.1% [wt/vol] and ammonium acetate 50% [wt/vol]) were used for complexing Fe(II), and iron(II) sulfate (0–525 μM) was used as a reference. For protein quantification, 200 µL of 1 M NaOH was added to 800 µL of the initial cell suspension and heated to 95°C for 10 min for cell lysis. Roti-Quant (Roth, Germany) was used to determine protein concentration with BSA (0–100 μg/mL) as a standard. The absorbance for Fe(II) and protein quantification was measured using a microplate reader (iMark Microplate Reader, Bio-Rad, Germany) at 595 nm.

### Whole-cell pull-down assay

To screen strains for successful MtrC secretion, a whole-cell pull-down assay was developed using SNAP-Capture Magnetic Beads (New England Biolabs) to selectively immobilize and magnetically separate SNAP-tagged proteins. Cells expressing and secreting MtrC_snap_ have a C-terminal SNAP tag attached to MtrC, which was hypothesized to be accessible on the cell surface after maturation and secretion of the mature MtrC. Thereby, it can bind to the corresponding beads added to the cells and thus can be used to separate those cells from cells that do not secrete MtrC_snap_. Magnetic beads were washed twice with buffer (50 mM HEPES, 300 mM NaCl, pH 8) prior to use. The investigated strains were mixed in a 1:1 ratio, and subsequently, 500 µL of cells OD_600_ = 2 were mixed with 50 µL of beads in LB medium. Beads containing covalently bound cells and non-bound cells were then separated using a magnetic separation rack. To remove all non-bound cells, beads were washed twice with buffer (50 mM HEPES, 300 mM NaCl, pH 8).

### Quantitative real-time PCR

To compare relative cell counts, bacterial suspensions heated to 98°C for 10 min served as templates. SsoAdvanced Universal SYBR Green Supermix (BioRad) was used according to the manufacturer’s instructions. Standards with defined cell numbers were prepared for calibration. Quantitative PCR (qPCR) was performed in a C1000 Thermal Cycler with CFX96 Real-Time System (BioRad) and analyzed using BioRad CFX Manager software. Primers used for qPCR are listed in Table S1 (numbers 26–33).

## RESULTS

In this work, the functionality of a putative minimal protein set for EET from the native exoelectrogenic organism *S. oneidensis* transplanted to *E. coli* was investigated. For this purpose, the different modules that are so far known to be part of the minimal protein setup, necessary for EET, were introduced step by step and thereby systematically analyzed. In total, six *E. coli* strains with different configurations regarding the heterologously expressed *c*-type cytochromes/*β* barrel proteins were investigated: *E. coli* WT, *E. coli* CymA (28), *E. coli* CymA STC, *E. coli* CymA STC MtrAB^s^, *E. coli* CymA STC MtrAB, and *E. coli* CymA STC MtrCAB (see Table 1 for details). The last strain thus possesses the putative complete minimal set of proteins that, according to current literature, enable the organism to reduce insoluble extracellular electron acceptors, while the CymA STC MtrAB^s^ strain should be able to reduce soluble extracellular electron acceptors at rates around 80% of the strain with the complete minimal set if results from *S. oneidensis* could be directly transferred to *E. coli* (22). *E. coli* CymA STC MtrAB was expected to be comparable to an *S. oneidensis* ΔOMC strain lacking the genes for all outer membrane cytochromes (43).

### AQDS reduction rates with different protein configurations differ largely between *S. oneidensis* and *E. coli*

**Fig 2:**
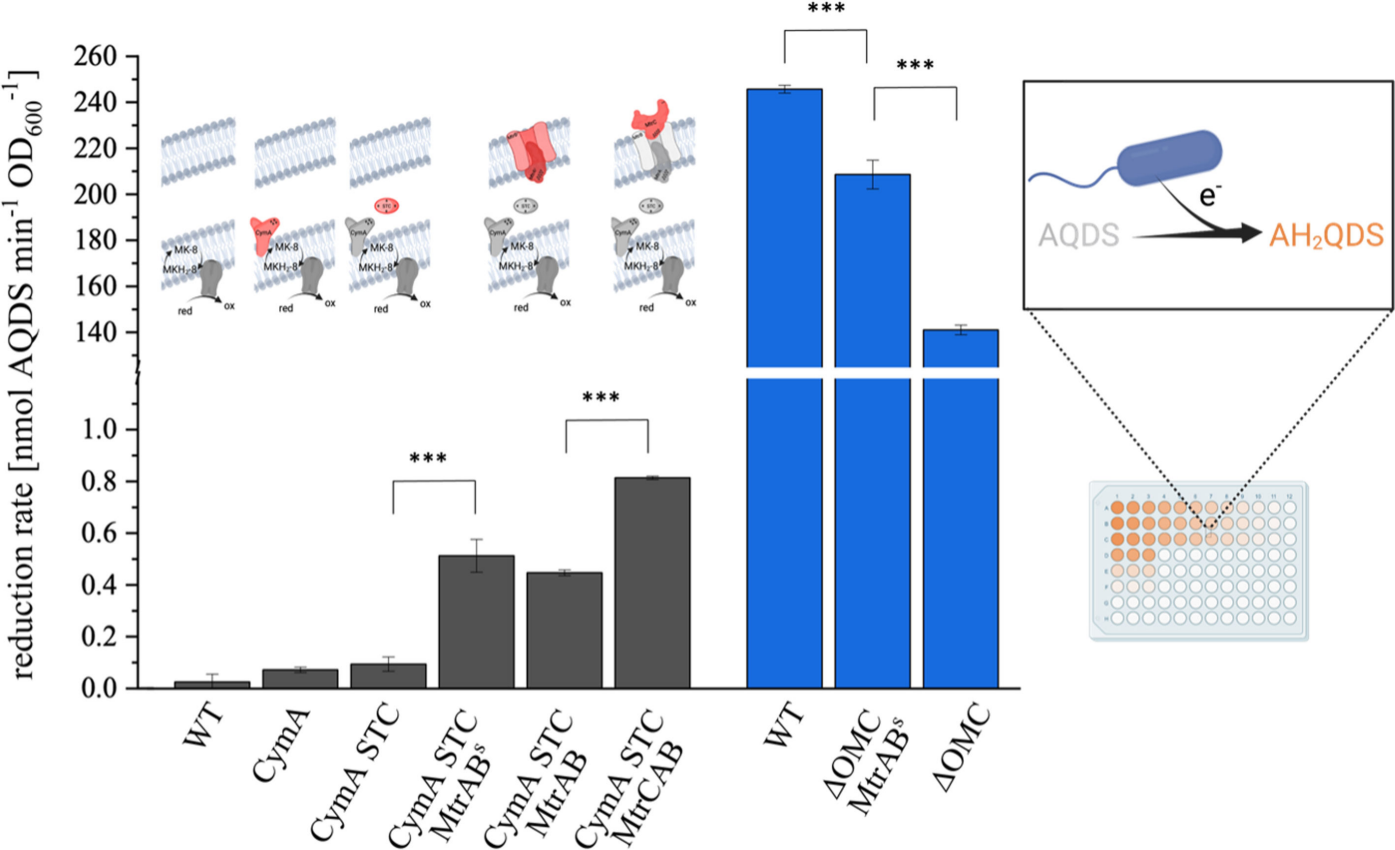
Maximum AQDS reduction rates of different *E. coli* and *S. oneidensis* strains. In gray (*E. coli*) and blue (*S. oneidensis*), the respective AQDS reduction rates are plotted in nmol AQDS min^−1^ OD_600_^−1^. The experiments were performed as cell suspension assays with OD_600_ = 6 (*E. coli*) and OD_600_ = 3 (*S. oneidensis*). The values shown are normalized to a uniform cell count. See text for explanations of individual strains. The mark *** indicates a significant difference in the respective reduction rate (unpaired *t*-test, *P* < 0.001). The scheme above shows the differences in the investigated *E. coli* expansion stages with regard to relevant added proteins in the inner and outer membrane as well as in the periplasm. On the right-hand side, the values for the reaction of biological AQDS reduction are shown. Reduction of AQDS to AH_2_QDS is accompanied by a change in color (from bright yellow to dark orange), which was quantified via a microplate reader.

The performance of the EET in *E. coli* and *S. oneidensis* was examined across all genetic engineering-based development stages via an AQDS reduction assay (Fig. 2). As a comparison, *S. oneidensis* strains (WT, ΔOMC, and the ΔOMC MtrAB^s^ suppressor mutant [Table 1]) were evaluated for their AQDS reduction rates. Of note, previous results revealed that AQDS in *S. oneidensis* was to a large extent reduced by cell surface cytochromes, a result that was also corroborated in this study (22). Hence, the assay is suitable for comparison of reduction rates based on periplasmic and cell surface-based reduction reactions. *E. coli* DH5αZI WT achieved a maximum reduction rate of 0.025 ± 0.031 nmol AQDS min^−1^ OD_600_^−1^. *E. coli* CymA showed a slightly increased maximum reduction rate of 0.072 ± 0.01 nmol AQDS min^−1^ OD_600_^−1^, which was further increased to 0.095 ± 0.023 nmol AQDS min^−1^ OD_600_^−1^ by co-expression of STC. *E. coli* CymA STC MtrAB^s^ is comparable to the *S. oneidensis* ΔOMC MtrAB^s^ suppressor mutant in terms of expressed proteins, so far known to be necessary for EET. Here, the *E. coli* strain achieved a maximum AQDS reduction state of 0.514 ± 0.064 nmol AQDS min^−1^ OD_600_^−1^, and the corresponding *S. oneidensis* strain achieved a maximum AQDS reduction state of 209 ± 6.24 nmol AQDS min^−1^ OD_600_^−1^. *E. coli* CymA STC MtrAB has an equivalent protein configuration relevant for EET comparable to the *S. oneidensis* ΔOMC strain. In the AQDS reduction assay, the two strains reached maximum rates of 0.448 ± 0.011 (*E. coli*) and 141.32 ± 2.08 nmol AQDS min^−1^ OD_600_^−1^ (*S. oneidensis*), respectively. Finally, the *E. coli* CymA STC MtrCAB_1_, in terms of so far known minimal EET protein setup comparable to *S. oneidensis* WT, achieved a maximum reduction rate of 0.816 ± 0.007 nmol AQDS min^−1^ OD_600_^−1^, whereas the *S. oneidensis* WT showed a maximum reduction rate of 246.18 ± 1.69 nmol AQDS min^−1^ OD_600_^−1^. When the corresponding strains are compared, the maximum reduction rates of the *S. oneidensis* strains are considerably higher than those of the respective *E. coli* strains. Moreover, the experiments revealed that *E. coli per se* shows only very minor AQDS reduction, which is consistent with other studies on AQDS reduction in *E. coli* (28, 72).

Following the integration of the respective sets of *c*-type cytochromes and the analysis of AQDS reduction rates, we quantified the heme content in the periplasmic and membrane fractions of *E. coli* CymA STC MtrCAB and compared it to *S. oneidensis* to assess any potential correlation between heme concentration and reduction efficiency. The results indicate that the heme content in the periplasmic fraction of *E. coli* CymA STC MtrCAB was even slightly higher (107%) than that of *S. oneidensis* WT (100%). The *E. coli* CymA STC MtrCAB membrane fraction showed a lower heme content, with values of 9% of the heme content found in the *S. oneidensis* WT membrane (100%).

### Heterologous expression of the TIISS genes is necessary for comparable MtrC exposure at the cell surface compared to *S. oneidensis*

**Fig 3:**
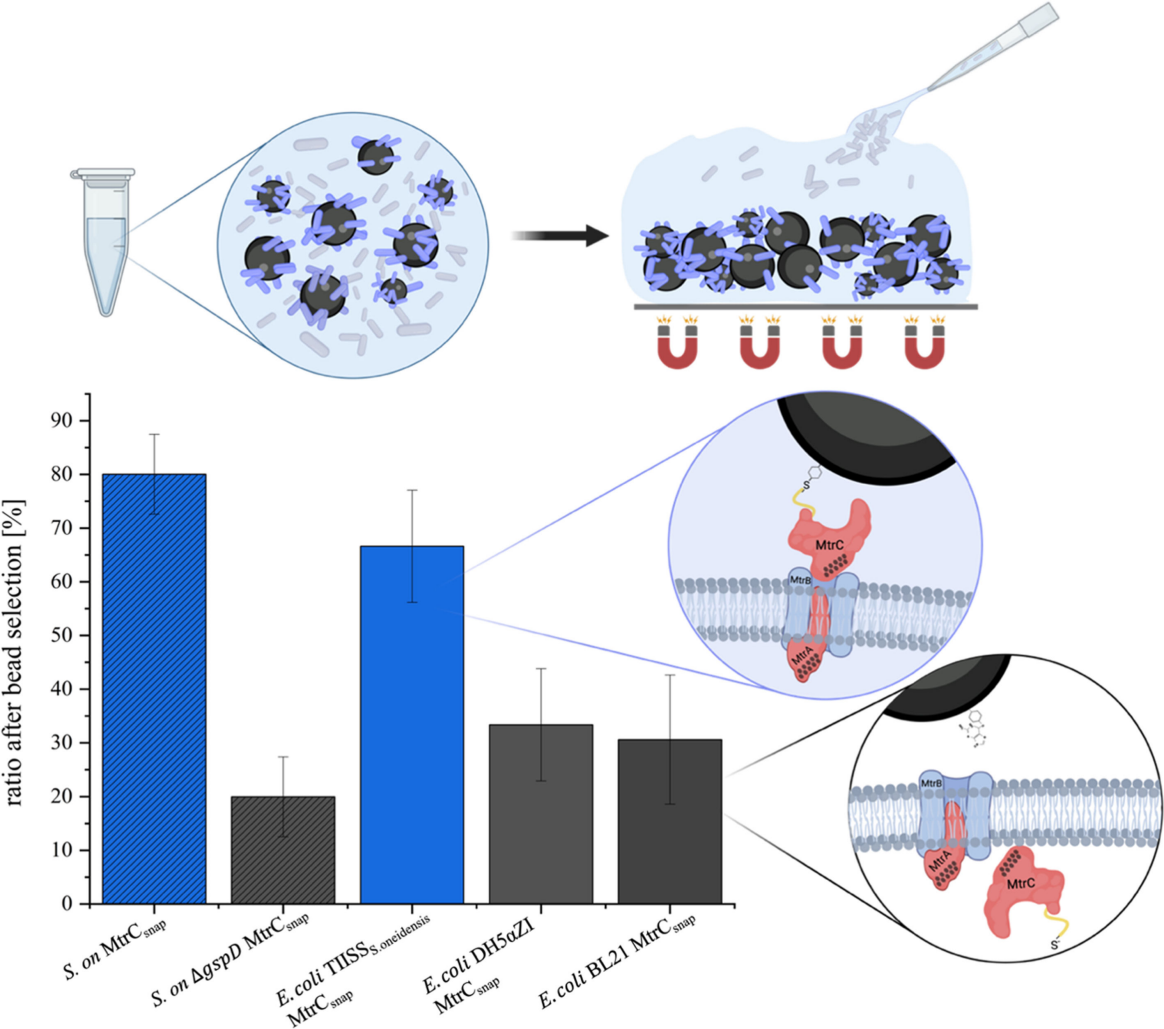
Ratio of investigated strains after pull-down using SNAP-Capture magnetic beads. Shown are the percentages of three pull-down assays with two strains each. *S. on* is used as the abbreviation for *S. oneidensis*. Shaded are the ratios of *S. oneidensis* WT (blue, *n* = 2) and *S. oneidensis ΔgspD* (gray, *n* = 2), both expressing MtrC_snap_. Shown without hatching are the ratios of *E. coli* DH5αZI TIISS*_S.oneidensis_* (blue, *n* = 5), *E. coli* DH5αZI (gray, *n* = 5), and *E. coli* BL21 (gray, *n* = 3), each expressing MtrC*_snap_*. The difference in abundance of all strains with functional TIISS*_S.oneidensis_* to strains without functional TIISS*_S.oneidensis_* after pull-down assay was significant at *P* < 0.01 (unpaired *t*-test). Above and right-hand side of the bars, a schematic overview shows the pull-down procedure and the mechanism of MtrC_snap_ binding to SNAP-Capture magnetic beads as described in the text.

Outer membrane cytochromes such as MtrC must pass through both the inner and outer membranes of *S. oneidensis* and *E. coli*, respectively. Secretion of proteins from the periplasm through the outer membrane is catalyzed by the type II secretion system (TIISS) encoded by the *gsp* genes. In *S. oneidensis* mutants with deletions in the most important components of the TIISS, namely the secretin GspD forming the secretion pore in the outer membrane, the secretion ATPase in the inner membrane, as well as GspE, the major pseudopilin in the pseudopilus, no MtrC can be detected on the outside of the outer membrane (42). Since various studies showed the high specificity of the TIISS, we investigated whether insufficient secretion of MtrC causes the observed losses in reduction rates between *E. coli* and *S. oneidensis* (73). Although heterologous expression of MtrC is possible in *E. coli* (see Fig. S2) and linkage of the corresponding heme groups in the periplasm is also successful, studies suggesting a functional transplantation of the EET chain never assessed the MtrC orientation within the outer membrane (54, 55). Therefore, we aimed to investigate if MtrC is correctly localized via the *E. coli* TIISS and, if not, whether expression of the TIISS of *S. oneidensis* could lead to correct localization of MtrC also in *E. coli*. To this end, the entire operon of TIISS genes from *S. oneidensis* was integrated into the genome of an *E. coli* strain lacking a functional endogenous TIISS (*E. coli* DH5αZI Δ(*napC-F*) Δ(*frdA-D*)::P_tet__*cymA*), and the influence of expression on the localization of MtrC was subsequently investigated. To provide optimal control of gene expression, the operon was divided into three clusters*—gspDEFC*, *gspHIJK,* and *gspGLMN*—and the genes were ordered according to their native transcription strength in *S. oneidensis* under iron-reducing conditions (see Fig. S3) (21). The resulting strain *E. coli*_TIISS_ was transformed with pEC86 as well as pBAD-*mtrCsnap_pilD*. The putative type IV prepilin peptidase PilD was co-expressed, since the activity of the TIISS and thus a secretion of outer membrane cytochromes in *S. oneidensis* depends on the presence of PilD (74). The heterologously expressed MtrC was SNAP-tagged (MtrC_snap_) to allow for subsequent localization studies, using the pull-down assay described above. This involved covalent binding and isolation of cells carrying MtrC_snap_ on the outer membrane using SNAP-Capture magnetic beads. Cells deficient in the secretion of MtrC do not carry an accessible SNAP-tag on the cell surface, and we hypothesized that these cells cannot bind to the beads. Quantification of cells bound to the beads was achieved using quantitative PCR. We mixed the two strains prior to adding the beads in a 1:1 ratio. The ratio of each strain was validated via quantitative PCR. We then performed the pull-down assay by adding the SNAP-Capture magnetic beads to the mixed culture and quantified the ratio of the two strains bound to the beads. For validation, two *S*. *oneidensis* strains (WT pBAD-*mtrC_snap_* and *ΔgspD* pBAD-*mtrC_snap_*) were first examined. This resulted in the ratios of 80.04% ± 7.45% (*S. oneidensis* WT pBAD-*mtrC_snap_*) to 19.96% ± 7.45% (*S. oneidensis ΔgspD* pBAD-*mtrC_snap_*) with corresponding *E. coli* ratios of 66.62% ± 10.45% (*E. coli* Dh5αZI_TIISS_ pBAD-*mtrC_snap_*) to 33.38% ± 10.45% (*E. coli* Dh5αZI pBAD-*mtrC_snap_*) and 30.6% ± 12.02% (*E. coli* BL21 pBAD-*mtrC_snap_*) shown in Fig. 3. Hence, similar results were achieved between control and functional TIISS expression in *S. oneidensis* and *E. coli*. As the *S. oneidensis ΔgspD* mutant is unable to export MtrC, we hypothesize that the cells that bound to the beads without expressing a functional TIISS interacted in an unspecific manner and that this is not due to partial export of MtrC.

**Fig 4:**
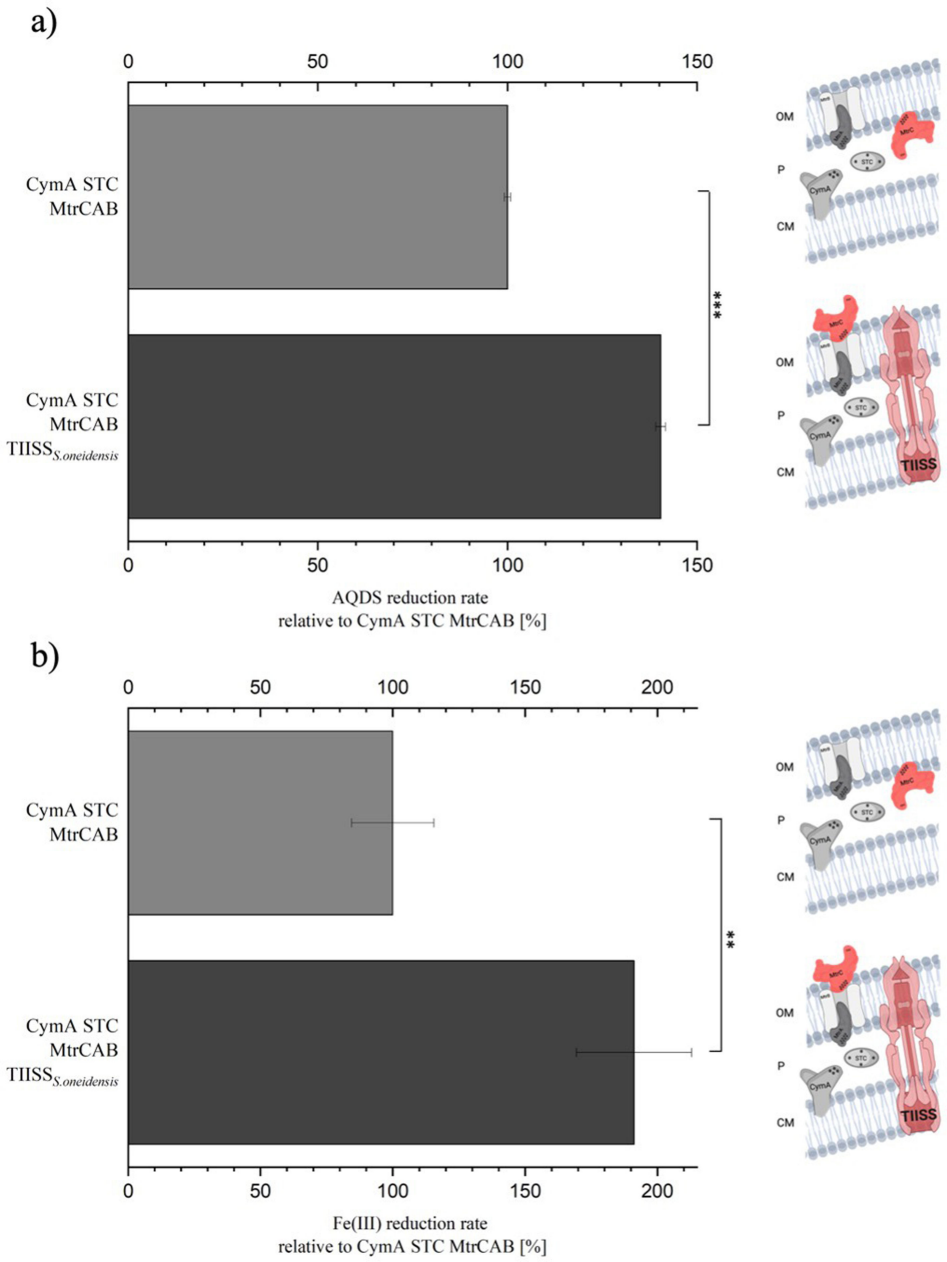
Relative AQDS and Fe(III)-citrate reduction rate of the *E. coli* TIISS_*S.oneidensis*_ strain. In (**a**), the relative AQDS reduction is shown. In dark gray, the reduction rate of the *E. coli* CymA STC MtrCAB TIISS_*S.oneidensis*_ strain is shown in percent. In light gray, the reduction rate of the *E. coli* CymA STC MtrCAB strain is shown in percent. The difference between *E. coli* TIISS_*S.oneidensis*_ and the control strain is significant (unpaired *t*-test, *P* < 0.001). In (**b**), the relative Fe(III) reduction is shown. In dark gray, the reduction rate of the *E. coli* CymA STC MtrCAB TIISS_*S.oneidensis*_ strain is shown in percent. In light gray, the reduction rate of the *E. coli* CymA STC MtrCAB strain is shown in percent. The difference between *E. coli* TIISS_*S.oneidensis*_ and the control strain is significant (unpaired *t*-test, *P* < 0.005). The scheme on the right-hand side shows the differences of the investigated strains with regard to relevant proteins in the inner and outer membrane as well as in the periplasm.

Next, we investigated whether the export of MtrC_snap_ to the cell surface also leads to an improved AQDS reduction rate. *E. coli* DH5αZI TIISS*_S.oneidensis_* reduced AQDS 1.4-fold faster compared to the progenitor strain without functional TIISS (Fig. 4a). This value is similar to the 1.7-fold increase measured when *S. oneidensis* WT and ΔOMC were compared (see Fig. 2). Of note, the expression of the snap-tagged version of MtrC is not expected to be correlated with lower extracellular reduction rates, as rescuing a *S. oneidensis* Δ*mtrC* mutant with heterologous expression of *mtrC*_(snap)_ was possible without a concomitant decrease in Fe(III)-citrate reduction rates (Fig. S4).

To exclude the effect of the suggested correct MtrC localization as a result of TIISS_*S.oneidensis*_ transplantation being due to potentially higher periplasmic AQDS reduction rates and to compare only extracellular reduction rates with previous experiments conducted with *S. oneidensis*, we conducted a similar cell suspension assay using Fe(III)-citrate. Previous results revealed that Fe(III)-citrate reduction in this assay is almost not detectable using an *S. oneidensis* mutant devoid of any outer membrane cytochrome (22). As expected, the effect on correct MtrC localization is even higher in this assay (Fig. 4b). The reduction rate increased twofold compared to 1.4-fold in the AQDS reduction test. *E. coli* CymA STC MtrCAB reduced 0.478 ± 0.074 nmol Fe(III)-citrate mg protein^−1^ min^−1^, and *E. coli* CymA STC MtrCAB TIISS*_S.oneidensis_* reduced 0.915 ± 0.104 nmol Fe(III)-citrate mg protein^−1^ min^−1^. Overall, the conducted experiments provide evidence that the correct localization of MtrC in *E. coli* K and B strains necessitates the specific activity of the type II secretion system of *S. oneidensis*. This is important as both B and K strains were used in previous studies and might differ in their type II secretion system expression and selectivity (28, 54–57).

### MtrB might be the bottleneck for ferric iron reduction in *E. coli*

**Fig 5:**
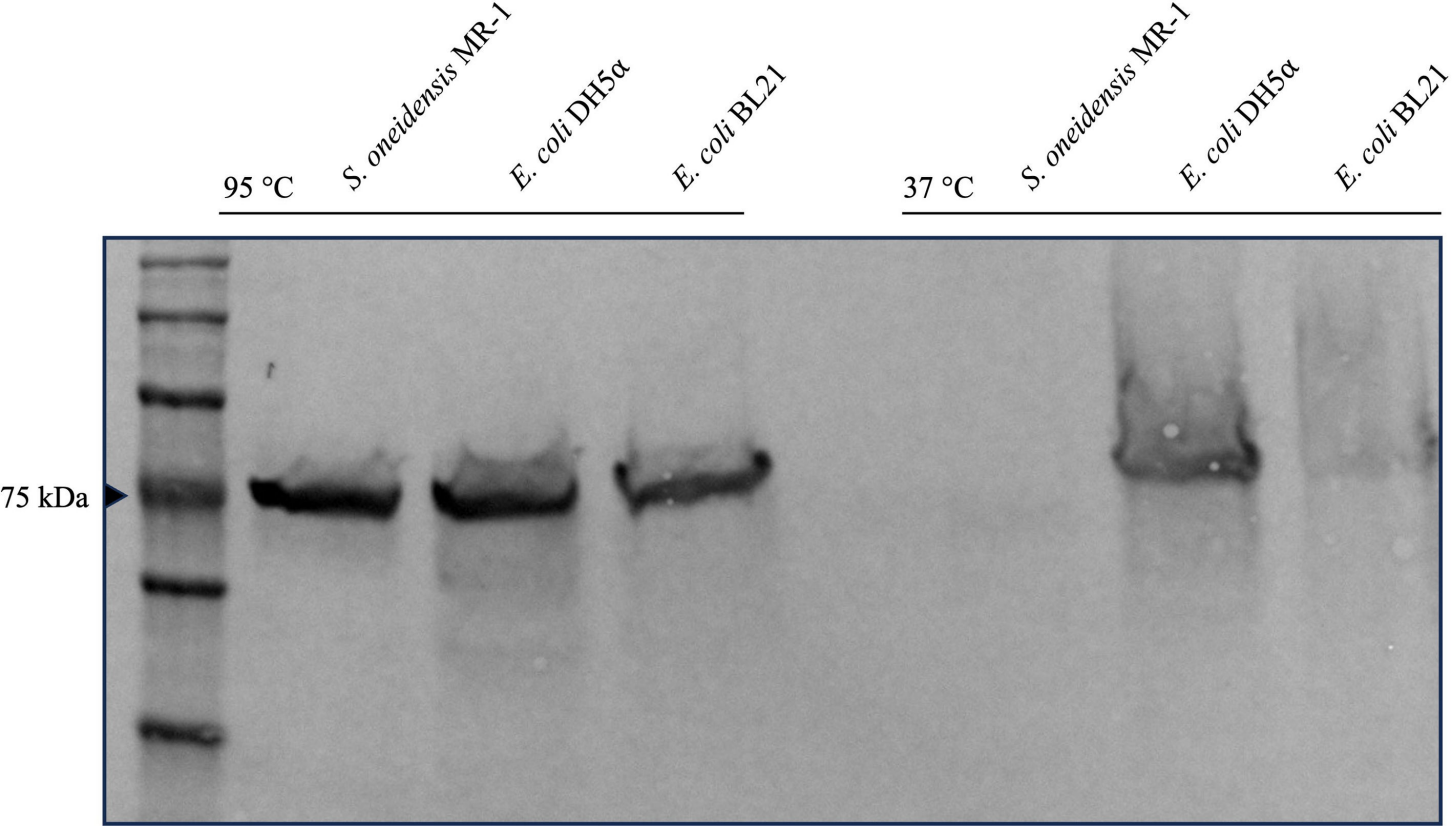
Immunodetection of MtrB in the membrane fractions of *S. oneidensis* MR-1, *E. coli* DH5αZI, and *E. coli* BL21, both expressing MtrAB. Shown is the immunodetection of MtrB via an anti-MtrB antibody in the membrane fractions. Prior to loading the SDS gel with each sample containing 100 µg total protein, the membrane sample was incubated either at 95°C or 37°C for 5 min.

Although we could increase the AQDS reduction rate and also the ferric citrate reduction rate by type II secretion system expression, the reduction rates for AQDS and ferric citrate are by far lower compared to *S. oneidensis*. Hence, we asked whether MtrB production in *E. coli* might lead to a loss in functionality. This hypothesis was raised as past studies suggested that MtrB is at least not guided through the periplasm comparable to canonical outer membrane *β*-barrel proteins such as OmpA (41). Since MtrB itself has no catalytic activity, it is not possible to develop a direct functionality-based assay. Instead, we were investigating the folding and unfolding behavior of MtrB expressed in *E. coli* compared to *S. oneidensis. β*-barrel proteins are rather stable and can retain their fold even in the presence of SDS. We compared heat-treated and non-heat-treated samples of MtrB in *S. oneidensis,* and surprisingly, we could not observe a band in Western blots of protein samples from the wild type that were not heat-treated, while a corresponding band could be observed in the identical but heat-treated samples (Fig. 5). This result suggests that the antibody binding site (AS)23–44 (70) is inaccessible if MtrB is in its native folded state. Next, we tested whether the observed pattern of antibody recognition in *S. oneidensis* could also be observed in *E. coli*. Thus, we tried the same experiment in an *E. coli* DH5αZI and an *E. coli* BL21 strain, respectively, expressing MtrAB using the same peptide-antibody (Fig. 5). Interestingly, MtrB was detectable in both strains, also in samples that were not heat-treated, even though the signal in the *E. coli* BL21 strain was not as strong as for the *E. coli* DH5αZI strain. Hence, the binding site for the antibody is accessible in all *E. coli* samples. In other words, MtrB probably does not fold the same way in *E. coli* as in *S. oneidensis* and might consequently be non-/less functional with regard to structuring the MtrCAB complex.

## DISCUSSION

### Heterologous expression of *S. oneidensis* electron transport proteins does not lead to competitive reduction rates in *E. coli*

Throughout the conducted experiments, it became evident that extracellular reduction rates in *E. coli* were not competitive compared to *S. oneidensis*. Heterologous expression of CymA alone or CymA and STC did not significantly increase the rate of AQDS reduction by *E. coli*. After integration of the MtrAB^s^ module spanning the outer membrane and proofing the expression of the respective proteins via heme stain (Fig. S1), an approximately 5.4-fold increase in AQDS reduction rate was obtained compared to the progenitor strain *E. coli* CymA STC. Since a large extent of AQDS reduction occurs outside the cell, it was expected that a significant increase in AQDS reduction would occur after integration of the MtrAB^s^ outer membrane module. However, it was surprising that an *E. coli* strain with the MtrAB module showed reduction rates without a significant difference from the MtrAB^s^ module. It is possible that AQDS diffuses more slowly into the periplasm in *E. coli* compared to *S. oneidensis* due to potential individual differences in lipopolysaccharide and extracellular polymeric matrix chemistry as well as porin content. Nevertheless, with a functional extracellular electron transport chain, at least similar values should be expected. This assumption is based on two observations. First, experiments with membrane fractions of *E. coli* cells overexpressing CymA revealed that even twofold higher reduction rates compared to *S. oneidensis* membranes could be achieved (28). Therefore, electron transfer into the periplasm is likely not the limiting factor. Second, a lower total respiratory or physiological capacity can also be excluded, as experiments using methylene blue as an electron shuttle show current densities in bioelectrochemical systems (BES) that are even higher than those compared to standard *S. oneidensis* BES experiments (75, 76). Hence, a lack of correctly folded MtrB might limit MtrAB^s^ electron transfer activity. Some evidence for this was provided by the experiments conducted here. Still, it could be possible that the expressed extracellular electron transport chain operates with similar activities in *E. coli,* but that the overall rates per cell are different due to lower protein concentration. While the expression of CymA, STC, MtrA, and MtrC in *E. coli* leads to an even higher concentration of heme protein in the periplasm, the concentration in the outer membrane was only 9%. It is important to note that *S. oneidensis* WT not only expresses MtrC in significant amounts in the outer membrane, but also the MtrA homologs as well as five other outer membrane cytochromes, of which at least OmcA is expressed to a considerable extent (45, 77). Hence, regarding the heme content in the outer membrane, reduction rates of at least 9% compared to *S. oneidensis* should be reached, which is by far not the case.

### MtrC and MtrB are so far overlooked bottlenecks regarding transferring the *S. oneidensis* extracellular electron transport chain to *E. coli*

Even though the components of the putative minimal electron transport chain from *S. oneidensis* were heterologously expressed in the *E. coli* strain, we could not reach competitive electron transfer rates compared to comparable *S. oneidensis* strains. MtrA, B, and C were in all previous studies revealed to be the most important proteins for extracellular electron transfer in *S. oneidensis*, as mutants in either one of the corresponding genes showed only minor electron transport rates so far, while mutants in other genes were often shown to be compensated by other proteins with overlapping function (18, 78). MtrA expression was shown before in *E. coli,* and its correct localization and maturation were revealed as well (29, 37). MtrC expression was shown, but its correct localization was often not assessed. Also, MtrB could be expressed in *E. coli*, but here it is not clear whether this protein is functional, as direct tests for activity are not possible, but it will depend on the formation of a functional complex with MtrC and MtrA. Due to this lack of information, we conducted a set of experiments to provide evidence for the functionality of MtrC and MtrB in *E. coli*.

Our analysis revealed that MtrC is, if at all, only partially exported through the outer membrane due to the specificity with which the TIISS detects the fold of targets for export. This specificity has been shown, among others, for the Pul system of *Klebsiella oxytoca*, which is unable to recognize proteins from the closely related γ-proteobacteria *Pseudomonas aeruginosa* or *Erwinia chrysanthemi* to secrete proteins (79, 80). Solving this issue by co-expressing the TIISS increased extracellular reduction rates, but the values were still far below what would be expected. Although not direct proof, the conducted experiments with MtrB give at least evidence for a different fold of MtrB in *E. coli* compared to *S. oneidensis*. Although it was possible to detect MtrB via western blot analyses in the past, these results were throughout gained via conventional sample preparation (e.g., 41, 54, 70). In this study, we could demonstrate that MtrB in *S. oneidensis* cannot be detected by a peptide antibody if the sample was not heat-treated prior to loading onto the SDS-gel. In contrast, we could demonstrate that MtrB in *E. coli* can be detected by the same peptide antibody regardless of whether the sample was or was not heat-treated prior to loading onto the SDS-gel. It is important to emphasize that the detected band in *E. coli* BL21 was weaker than that observed in *E. coli* DH5α. Additionally, we would like to point out that the intensity of the band at 37°C in *E. coli* DH5α was lower than that at 95°C. However, at this stage, it is not possible to determine whether the reduction in intensity follows a similar trend in both strains, as quantification was not feasible due to the indistinct nature of the bands. While we cannot yet fully exclude the possibility of differences in the folding and incorporation of MtrB between B and K strains, our findings indicate that in both strains, a significant proportion of MtrB might not be correctly folded.

The peptide antibody used in this study binds to an N-terminal region (AS)23–44 (70), which is close to a potential disulfide bond within MtrB, formed between cysteine 42 and 45 (70, 81). Wee et al. revealed via site-directed mutagenesis that *S. oneidensis* MtrB C42A mutants lost their ability to reduce ferric iron, while C45A mutants behaved like the wild type. This led the authors to the hypothesis that C42 might be involved in heterologous disulfide bond formation (81). Interaction with other proteins or binding partners could lead to a different 3D structure compared to the same protein without disulfide bonds. Unfortunately, the MtrB structure from *S. baltica* does not include the N-terminal CxxC motif. Therefore, it is not possible to see a potential engagement of C42 in a heterologous disulfide bond (40).

If we assume that MtrB will not have the same conformation in an *E. coli* outer membrane compared to an *S. oneidensis* outer membrane, we would be able to explain why attempts to express the MtrCAB conduit in *E. coli* always led to strains that had a by far lower ferric iron reduction rate compared to *S. oneidensis* (54). Also, if MtrB folding is not correct, even production of the suppressor module MtrAB^S^ cannot have a similar effect in *E. coli* compared to *S. oneidensis*, which is exactly what could be observed here. Hence, it might be necessary to add one or more so far unknown other factors from *S. oneidensis* for the formation of a functional MtrCAB complex, to reach comparable electron transfer rates in the future. Notably, besides possibly missing folding factors, structural distinctions in the membrane and lipopolysaccharide (LPS) composition between *E. coli* and *S. oneidensis* need to be considered, as well as factors for MtrB maturation. In *E. coli*, the outer membrane is characterized by a high LPS content, contributing to a negatively charged surface. In contrast, *S. oneidensis* possesses a multitude of outer membrane cytochromes, which are lipoproteins and thus increase the lipoprotein content in the outer membrane (82). The differences in LPS could significantly impact the membrane properties, including charge, permeability, and interactions with external molecules, potentially influencing the folding and function of membrane proteins like MtrB (83). In *E. coli*, the LPS structure typically comprises the three regions: lipid A, the core oligosaccharide connected to lipid A, and the O-antigen. The latter is missing in *E. coli* B strains, which results in a rough LPS phenotype (84). In contrast, *S. oneidensis* exhibits a kdo modification in its core oligosaccharide, which is believed to enhance outer membrane integrity, and compared to *E. coli* K strains, the O-antigen is missing (85, 86).

### Conclusions

In this work, the successful secretion as well as correct localization of the terminal reductase MtrC upon heterologous expression in *E. coli* could be demonstrated. For this purpose, the TIISS from *S. oneidensis* was transplanted and heterologously expressed in *E. coli*. This was accompanied by a 1.4-fold and twofold increase in the AQDS and ferric citrate reduction rate, respectively. Furthermore, this study revealed that misfolding of the *β*-barrel protein MtrB in *E. coli* might further hamper the overall EET performance with regard to reduction rates. In order to address this possible bottleneck, the investigation of further necessary genetic adaptations of *E. coli* should be considered.

## SUPPLEMENTAL MATERIAL

Supplemental material aem.00685-25-s0001.docx Table S1, primer list; Fig. S1 to S4, additional experimental details like heme stains, transcription pattern, and ferric citrate reduction assay.

ASM does not own the copyrights to Supplemental Material that may be linked to, or accessed through, an article. The authors have granted ASM a non-exclusive, world-wide license to publish the Supplemental Material files. Please contact the corresponding author directly for reuse.
