# Supplementary material for: Identification of factors limiting the efficiency of transplanting extracellular electron transfer chains in Escherichia coli
Source: Appl Environ Microbiol. 2025 May 13;91(6):e00685-25. doi: 10.1128/aem.00685-25 (PMC12175506; doi:10.1128/aem.00685-25)
Supplement: Supplemental material — Table S1, primer list; Fig. S1 to S4, additional experimental details like heme stains, transcription pattern, and ferric citrate reduction assay. [file aem.00685-25-s0001.docx]

**Supplementary Material**

Table S1: Primers, used in this study.

| Nr. | Name | Sequence |
| --- | --- | --- |
| 1 | P_ara__for_ol_pAH95 | TCCTGACGGATGGCCTTTTTGCGTGGCCAGTGCCAAGCTTGCATGCCTGCAAAGAAACCAATTGTCCATATTG |
| 2 | P_ara__rev_ol_MtrA | GTGATGGTCAGTGCCGGCAGCAGGTTTTTCATTTTGAGGCAGTTCTTCATGGTATGTATATCTCCTTCTTAAAG |
| 3 | *mtrB*_rev_ol_pAH95 | TCACTGATCAGTGATAAGCTGTCAAACATGAGAATTCGAGCTCGGTACCCTTAGAGTTTGTAGCTCATGC |
| 4 | P_lac__for | TTATCAAAAAGAGTGTTGACTTG |
| 5 | *mtrC*_for_ol_pBADNcoI | TTTGGGCTAGAAATAATTTTGTTTAACTTTAAGAAGGAGATATACATACCATGATGAACGCACAAAAATCA |
| 6 | *mtrC*noStop_rev-Linker | ACATTCACGACGTTCTTTAGTTTCAGATTGGTGACAAGAAACCATTTTCACTTTAGTGTGATCTG |
| 7 | for_snap_ol-linker_*mtrC* | GTTTCTTGTCACCAATCTGAAACTAAAGAACGTCGTGAATGTATGGACAAAGATTGCGAAATG |
| 8 | Snap_rev | TTATCCCAGACCCGGTTTA |
| 9 | RBS_*pilD*_for_ol_Snap | GCTGCTGGCCCATGAAGGTCATCGTCTGGGTAAACCGGGTCTGGGATAATACATTGAACAATATAATTAAGTAAG |
| 10 | *mtrC*_for_ol_RBS-*cctA* | CTGAAGAAGTAAAGCACTAAATAAAAGGAGTGAAGGGAAATATGGGCAACGCACAAAAAT |
| 11 | rev_*cctA*_OL_pMAL | AAACGACGGCCAGTGCCAAGCTTGCCTGCAGGTTATTTTTTCAGAACAGATGCGC |
| 12 | KI_rev_ol_pAH95 | TCACTGATCAGTGATAAGCTGTCAAACATGAGAATTCGAGCTCGGTACCCTCACTGCGGCAGAGAAAAC |
| 13 | P_lac__KI_for_ol_pAH95 | TCCTGACGGATGGCCTTTTTGCGTGGCCAGTGCCAAGCTTGCATGCCTGCATTATCAAAAAGAGTGTTGACTTGT |
| 14 | KII_*galK*_for | AAACGGGAGTTAAACATATCAGAGACGCCTCTGATTTGGCAAAGATTTACCATGTGGGAGTTTATTCTTG |
| 15 | KII_*galK*_rev | AGAAATTTCGAAAGGTAAAATATCCTTGTCACATTCGTTTGCAAAGGAAGGCCAGTGCGGGAGTTTCGTT |
| 16 | KII_500_up_for | TAGGGATAACAGGGTAATCATAATCAGGTCGCGTCCGG |
| 17 | KII_500_up_rev | GTTTAAACGTAAATCTTTGCCAAATCAG |
| 18 | KII_500_down_for | AAACGGGAGTTAAACATATCAGAGACGCCTCTGATTTGGCAAAGATTTACGTTTAAACCTTCCTTTGCAAACGAATGT |
| 19 | KII_500_down_rev | ATTACCCTGTTATCCCTATGACTTCATAATGCTCTTTG |
| 20 | KIII_*galK*_for | ATATTGAAAAAAATATCACCAAATAAAAAACGCCTTAGTAAGTATTTTTCCATGTGGGAGTTTATTCTTG |
| 21 | KIII_*galK*_rev | TTTTAATCCACACAGAGACATATTGCCCGTTGCAGTCAGAATGAAAAGCTGCCAGTGCGGGAGTTTCGTT |
| 22 | KIII_500_up_for | TAGGGATAACAGGGTAATTCGATTTCACTGTCGCCACC |
| 23 | KIII_500_up_rev | CCATGGGAAAAATACTTACTAAGGCG |
| 24 | KIII_500_down_for | ATATTGAAAAAAATATCACCAAATAAAAAACGCCTTAGTAAGTATTTTTCCCATGGAGCTTTTCATTCTGACTGCA |
| 25 | KIII_500_down_rev | ATTACCCTGTTATCCCTAATGGTTTTTTCAATCATCGC |
| 26 | Start_CymA_for | ATGAACTGGCGTGCACT |
| 27 | CymA_rev | CCAACAACCAGTAGCGC |
| 28 | Nuo_for | CAGTGATTGTAAGGTTTTTTTT |
| 29 | Nuo_rev | GATGACTTCAGTGGATGTTG |
| 30 | *gspD*_for | GCACCTACATCACCAATCC |
| 31 | *gspD*_rev | CACAATGAAAGAGGCTTCCTG |
| 32 | *mioC_*for | TATTCAAGTGCTTCTATTAG |
| 33 | *mioC_*rev | AAGAACTTCTACTCAACA |
| 34 | Gent^R^_for_ol_pEC86 | TGACACCCTCATCAGTGCCAACATAGTAAGCCAGTATACACTCCGCTAGCGCCTTGAACGAATTGTTAGGTGG |
| 35 | Gent^R^_rev_ol_ P_lac_ | GTATCATTGTTATCCGCTCACAAGTCAACACTCTTTTTGATAAGCGGAACCCCTATTTGTTTA |
| 36 | P_lac__rev_ol_*mtrA* | GTGATGGTCAGTGCCGGCAGCAGGTTTTTCATTTTGAGGCAGTTCTTCATTATTTTTCTTTCCCCTAACGC |
| 37 | *mtrB*_rev_ol_pEC86 | TCCAGGCCACTCATACCTTTCATATTGAGATCCAGCAAGATCACGTCGATTTAGAGTTTGTAGCTCATGCTC |


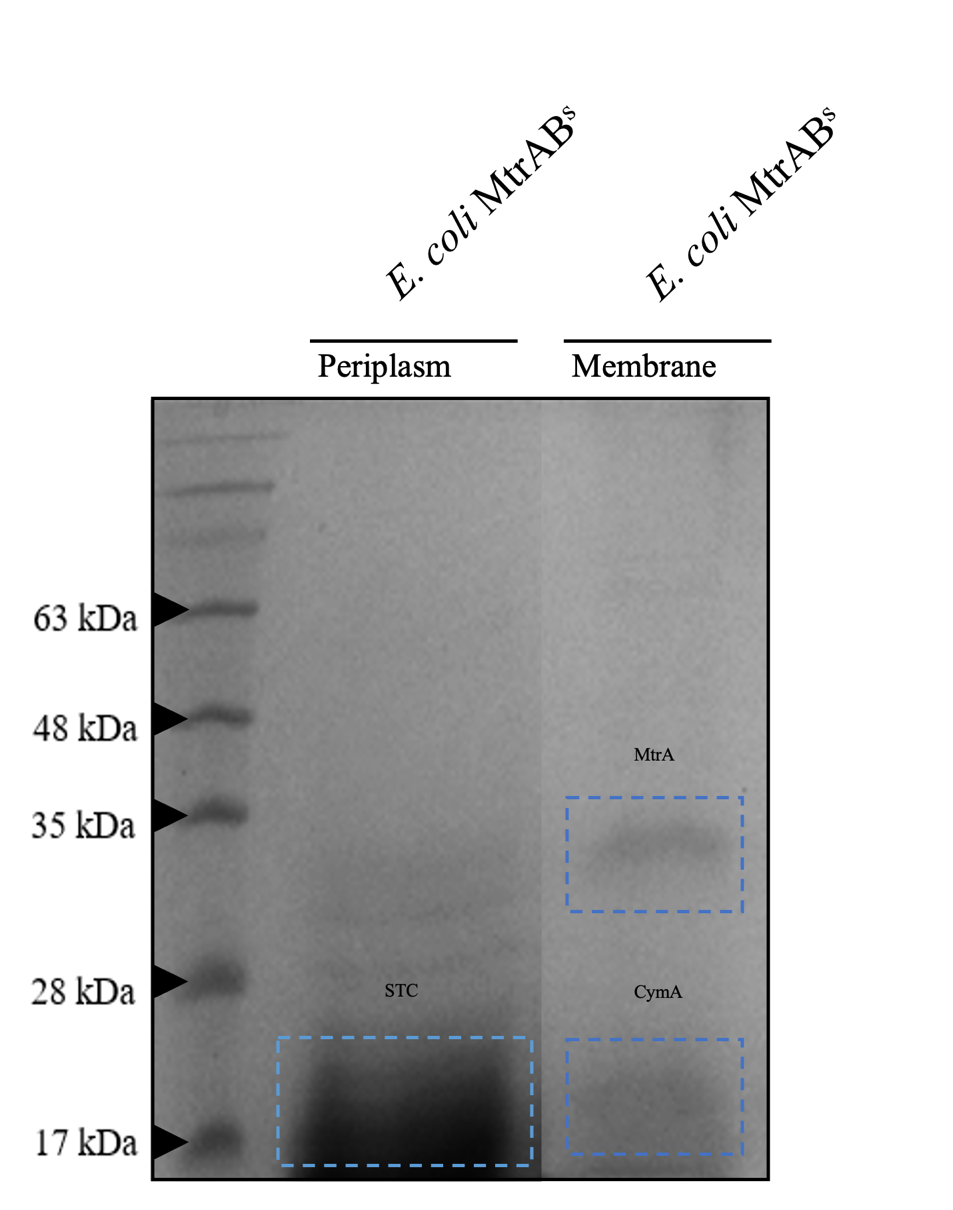


**Fig. S1: Heme staining of periplasm and membrane fractions of *E. coli*MtrAB^s^*:***100 μg (membrane) and 50 μg (periplasm) total protein was loaded onto the SDS gel. The fractions show bands correlating with the sizes of the heterologous *c-*type cytochromes, relevant bands were marked in blue.


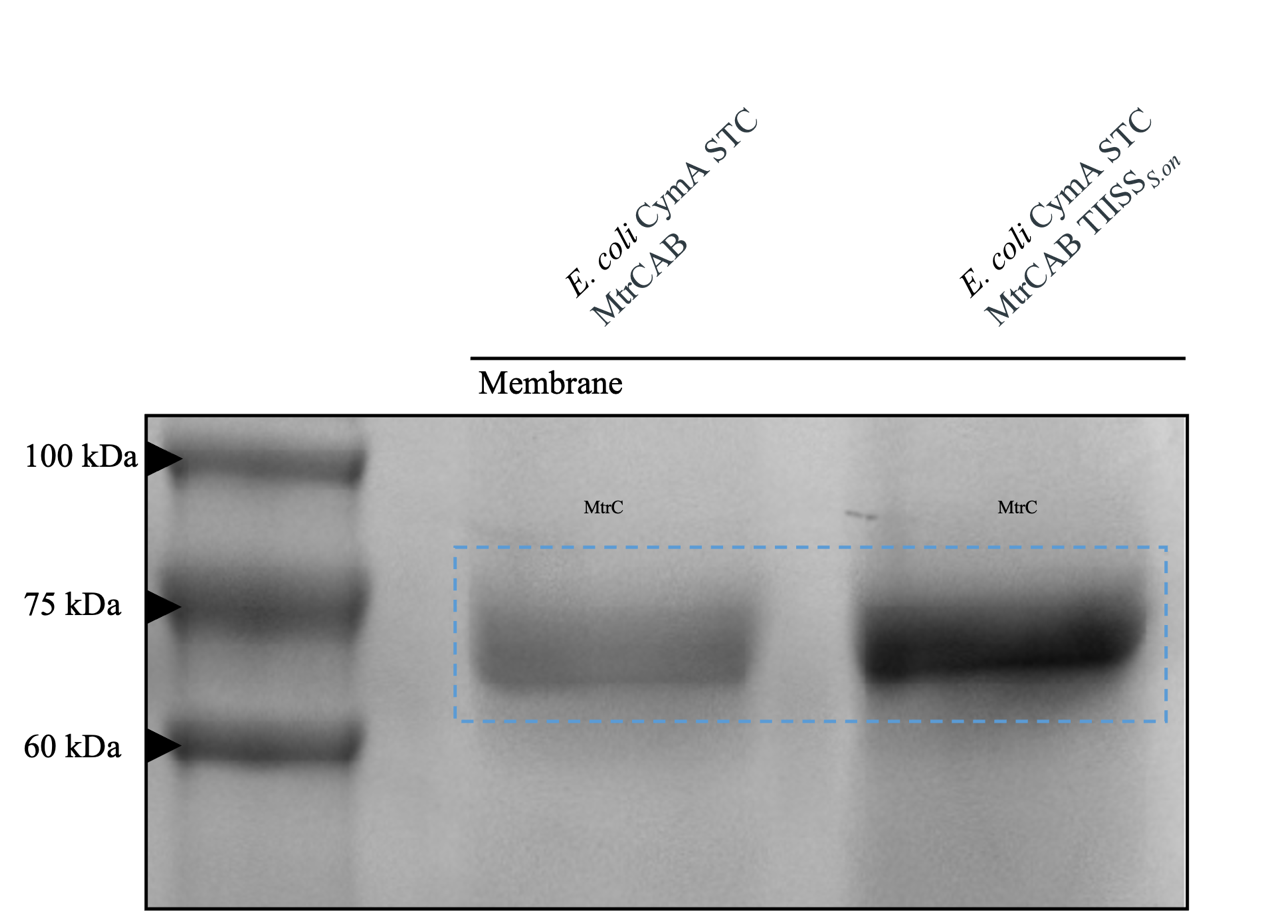


**Fig. S2: Heme staining of membrane fractions of *E. coli*MtrCAB and *E.* *coli.* MtrCAB TIISS*_S.on_*:**100 μg total protein was loaded onto the SDS gel. The fractions show bands correlating with the sizes of MtrC, relevant bands were marked in blue.


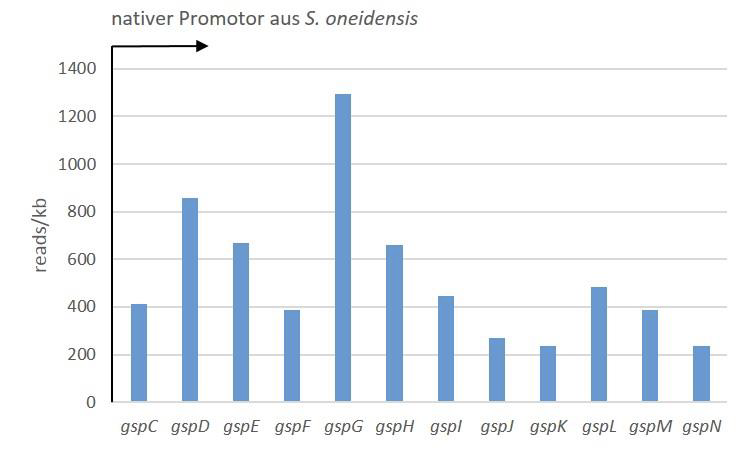

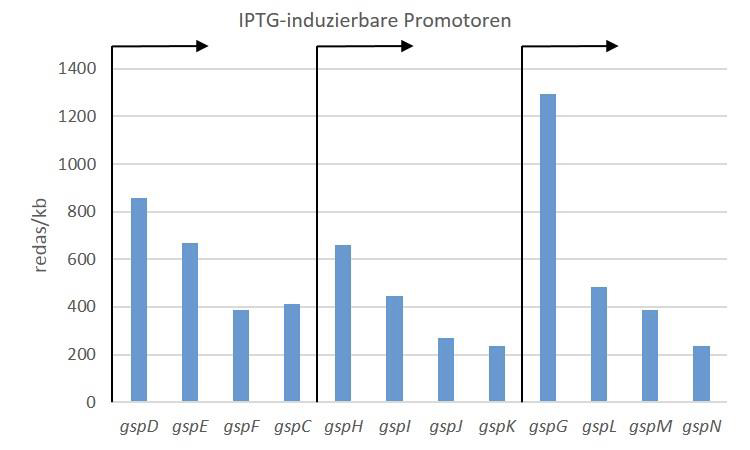


a

b

**Fig. S3: Transcription pattern of the *gsp* genes in *S. oneidensis* and re-arrangement for mimicking the pattern in *E. coli*:** **a** shows transcription pattern of the *gsp* genes in *S. oneidensis* MR-1 under anaerobic, iron-reducing conditions. Transcript levels are shown as gene length-corrected expression of each gene. **b** shows the re-arrangement for integration of the *gsp* genes into *E. coli* according to the native transcription strength. The *gsp* operon from *S. oneidensis* was divided into three individual operons under control of an IPTG-inducible promoter.

a

b


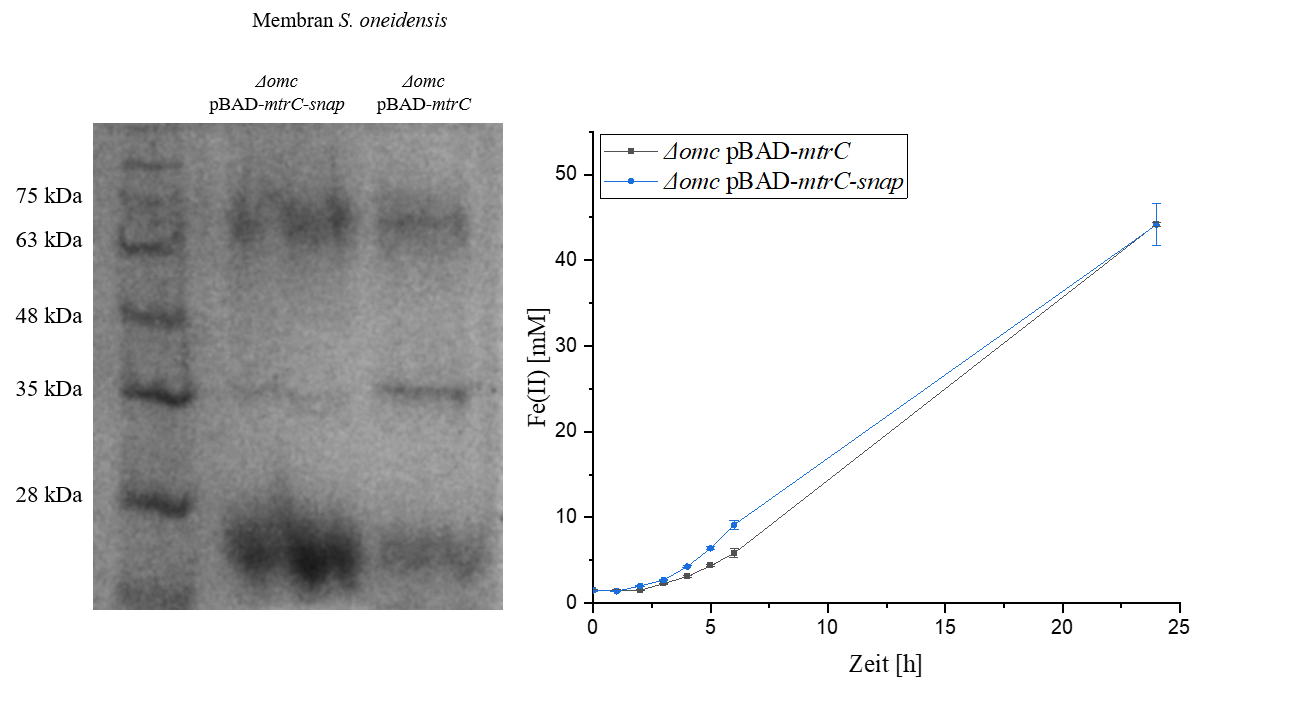


MtrC

time [h]

**Fig. S4: Investigation of the expression and functionality of MtrC_snap_:** In **a,** the heme group staining of the membrane fractions of *S. oneidensis* *Δomc* pBAD-*mtrC_snap_* and *S. oneidensis* *Δomc* pBAD-*mtrC* is shown. For each membrane fraction 100 μg total protein was applied. The induced fractions show bands that correlate with the sizes of the *c*-type cytochromes to be detected. The bands corresponding to the size of MtrC are marked in blue. In **b**, the increase of Fe(II) in a Fe(III)-citrate reduction assay is plotted over time. The strain expressing *mtrC_snap_* is shown in blue, the strain expressing *mtrC* is shown in gray.
